# Combining DCQGMP-Based Sparse Decomposition and MPDR Beamformer for Multi-Type Interferences Mitigation for GNSS Receivers

**DOI:** 10.3390/s17040813

**Published:** 2017-04-10

**Authors:** Qiang Guo, Liangang Qi

**Affiliations:** College of Information and Communication Engineering, Harbin Engineering University, Harbin 150001, China; guoqiang@hrbeu.edu.cn

**Keywords:** GNSS, multi-type interferences suppression, sparse decomposition, DCQGMP, MPDR

## Abstract

In the coexistence of multiple types of interfering signals, the performance of interference suppression methods based on time and frequency domains is degraded seriously, and the technique using an antenna array requires a large enough size and huge hardware costs. To combat multi-type interferences better for GNSS receivers, this paper proposes a cascaded multi-type interferences mitigation method combining improved double chain quantum genetic matching pursuit (DCQGMP)-based sparse decomposition and an MPDR beamformer. The key idea behind the proposed method is that the multiple types of interfering signals can be excised by taking advantage of their sparse features in different domains. In the first stage, the single-tone (multi-tone) and linear chirp interfering signals are canceled by sparse decomposition according to their sparsity in the over-complete dictionary. In order to improve the timeliness of matching pursuit (MP)-based sparse decomposition, a DCQGMP is introduced by combining an improved double chain quantum genetic algorithm (DCQGA) and the MP algorithm, and the DCQGMP algorithm is extended to handle the multi-channel signals according to the correlation among the signals in different channels. In the second stage, the minimum power distortionless response (MPDR) beamformer is utilized to nullify the residuary interferences (e.g., wideband Gaussian noise interferences). Several simulation results show that the proposed method can not only improve the interference mitigation degree of freedom (DoF) of the array antenna, but also effectively deal with the interference arriving from the same direction with the GNSS signal, which can be sparse represented in the over-complete dictionary. Moreover, it does not bring serious distortions into the navigation signal.

## 1. Introduction

The global navigation satellite system (GNSS) plays an increasingly important role in military and civil areas; however, the risk caused by the vulnerability of GNSS signals is getting more and more serious. Therefore, interference mitigation techniques are absolutely essential to ensure the reliability, accuracy and continuity of GNSS services. In terms of the characteristics of the GNSS, one way of enhancing its ability of mitigating radio frequency interferences is to improve the design of navigation satellites [1], for example optimizing the structure of signals and increasing the power of transmitters; but, these methods are too costly in terms of the design cycle and material resources. The other way is to improve the anti-jamming performance of GNSS receivers, which has attracted significant attention due to its low cost, high flexibility and great scalability.

The interference mitigation techniques for GNSS receivers can be classified into the time-domain, transform-domain, spatial and spatial-time processing [2]. On account of the characteristics of interferences in the time-domain and transform-domain, many methods have been extensively studied (e.g., [3,4,5,6]). The time-domain and frequency-domain processing methods are mainly used for suppressing fewer stationary narrow-band interferences. The time-frequency processing is one of the most powerful methods for suppressing the interfering signal, which has more concentrated energy distribution in the time-frequency domain than GNSS signals. For example, Chien et al. proposed to generate a reference signal by using wavelet-packet-transform-based adaptive predictors and subtracting the refined interfering signal from the received signal [7]. Additionally, it is effective to combat chirp interferences and single-tone interferences with a high interference-to-signal power ratio. However, when they are used to combat wideband interferers whose time-frequency characteristics are irregular (such as Gaussian interferers) or multiple interferences whose time and frequency characteristics are varied and vary rapidly in the frequency domain, their performance degrades seriously.

The space-based methods utilizing an antenna array are able to effectively mitigate narrowband and wideband interferences no matter what their time and frequency characteristics are. For example, the minimum variance distortionless response (MVDR) beamformer [8] is one of the most powerful spatial processing methods, which has a distortionless response for the desired signal while rejecting all interferences arriving from other directions. Additionally, the spatial processing has been mainly used for interference mitigation in GNSS applications [9]. According to the energy of GNSS signals in the receiver being less than that of the thermal noise, [10] drew the attention to mitigating interferences whose power is significantly higher than that of global position system signals by employing the minimum power distortionless response (MPDR) beamformer. Additionally, its effectiveness is doubtless as a verified method by practice. Nevertheless, the number of interferences that can be dealt with is limited to the number of antenna elements. Antenna design complexity increases with the number of elements, and so does the cost. In addition to that, given a limited physical space, placing several antenna elements close to each other could lead to undesired interactions, which will degrade the performance. In order to deal with more interferences with a limited number of antenna elements, the spatial temporal adaptive processing (STAP) is introduced [11]. By combining time and spatial processing, it is possible to increase the number of suppressed narrowband interfering signals without extra elements in the array. Although their superior advantages are obvious and desirable, the STAP may introduce cross-correlation function biases and distortions, which can result in inferior position estimates [11,12,13]. This is mainly because of the temporal part of filtering [14] and the non-linearity behavior of its frequency response [15]. These biases and distortions vary according to the direction of arrival (DOA) and the number of interfering signals. Additionally, each one of the distortions is unique for each desired signal. To obtain accurate position and time solutions, some methods reducing the distortions and biases have been studied [16,17]. They reduce the DoF of interferences mitigation and bring more complexity both in arithmetic and implementation.

In addition, since the jamming technology develops rapidly and the electromagnetic environment is increasingly complex, there may be multiple types of interfering signals presenting simultaneously rather than only one or one type of interfering signals in the receiving environment. Although existing interference mitigation methods are able to improve the performance of GNSS receivers in the presence of interferences, there are enormous challenges when they suppress multiple types of interferences: (1) the hardware or space costs are enormous; (2) when the DOA of the interference (especially for the wideband interference) is close to the GNSS signal, their performance degrades seriously; (3) the methods using both spatial and time-/frequency-domain processing may distort the GNSS signals.

To address the above problems, some cascaded multi-type interferences mitigation methods using frequency and spatial (or spatial-time) domains are introduced (e.g., [18,19]). These methods are “simple cascades” of existing techniques without considering the mutual overlaps of different signals. It is one of the obvious disadvantages that when the narrowband interfering signals fall in the wideband Gaussian interferences, the pre-stage methods may be invalid.

In the coexistence of multiple types of interfering signals, there may be overlaps among interfering signals in the time, frequency and spatial domains. It is an enormous challenge for existing methods to deal with multiple interferences. To address this problem, we introduce the signal sparse decomposition theory [20] into array signal processing. The sparse decomposition theory is gaining significant attention due to its advantages in digital signal processing and has been successfully applied to many fields, for example signal detection [21], clutter and jamming suppression [22]. Then, a cascaded multi-type interferences mitigation method combining improved DCQGMP-based sparse decomposition and the MPDR beamformer is proposed in this paper. In the first stage, the single-tone (multi-tone) and linear chirp interfering signals can be detected and canceled by utilizing an improved DCQGMP-based sparse decomposition according to their sparsity in the over-complete dictionary. In order to reduce the running time and detect interference signals that may fall in the other wideband interferences effectively, a novel design strategy of the over-complete dictionary and MP algorithm is proposed, and DCQGA [23] is used to approach the MP optimization problem. To solve the problems of slow convergence speed, low search precision and poor robustness in the traditional DCQGA, the encoding mode and rotation angle computing function are improved. In the second stage, the MPDR beamformer is adopted to suppress the residual interferences, which is able to enhance the steering gain of the DOA of GNSS signals while nullifying interferences. Finally, the performance of the improved DCQGA is verified by a simulation. Additionally, several simulation scenarios are considered to show the effectiveness of the proposed method for multi-type interferences mitigation, and the compared methods are the well-known MPDR beamformer [10] and the distortionless space-time processor [16].

## 2. Signal Model

The received signal should be an aggregate of GNSS signals, the receiver thermal noise and interfering signals. For the convention of description, suppose that there is only one GNSS signal contaminated by *K* interferences and the thermal noise. Additionally, they are independent of each other and adhere to the narrowband model. Then, for the *m*-th sampling time, the complex representation of the signal vector received by an arbitrary antenna array with *N* elements can be expressed as:

(1) $$\begin{array}{c} x\left(m\right)=\left[x_{1}\left(m\right)x_{2}\left(m\right)\cdots x_{N}\left(m\right)\right]=\mathrm{as}\left(mT_{s}\right)+\sum_{i=1}^{K}b_{i}J_{i}\left(mT_{s}\right)+\eta \left(mT_{s}\right) \end{array}$$

where $T_{s}$ is the sampling period, $J_{i},i=1,2,\cdots ,K$, indicates the i-th interfering signal and $b_{i},i=1,2,\cdots ,K$ is the corresponding steering vector. s denotes the GNSS signal, and η represents the receiver thermal noise. Additionally, **a**, representing the steering vector of the GNSS signal whose dimension is N×1, can be written as:

(2) $$\begin{array}{c} a={\left[e^{j\frac{2\pi }{\lambda }{\left(d\right)}^{T}o_{1}}\;e^{j\frac{2\pi }{\lambda }{\left(d\right)}^{T}o_{2}}\;\ldots \;e^{j\frac{2\pi }{\lambda }{\left(d\right)}^{T}o_{N}}\right]}^{T} \end{array}$$

where λ denotes the wavelength of received signals; d is a 3 × 1 unit vector representing the DOA of the GNSS signal; $o_{n}$, n=1,2,…,N is a 3 × 1 unit vector pointing to the n-th antenna element; ‘${\left(•\right)}^{T}$’ denotes transpose.

The interfering signal can be classed into different types according to the interferers generating it. In this paper, interferences are considered to be in the class of continuous wave interferences (CWI) and wideband Gaussian noise signals, which can be written as:

(3) $$\begin{array}{c} J\left(m\right)=\sum_{i=1}^{K1}J_{cw_{i}}\left(m\right)+\sum_{i=1}^{K2}J_{g_{i}}\left(m\right) \end{array}$$

where $J_{g_{i}}$ denotes a Gaussian interfering signal with zero mean and $\sigma_{i}$ variance; and K1, K2 represent the number of CWI and wideband Gaussian noise signals. In general, the CWI, $J_{cw_{i}}$, usually is a frequency-modulated signal with an almost constant amplitude. The linear chirp and single-tone (or multi-tone) signals, which are the main types of CWI [24], are considered in the following analysis. Their time-domain function can be expressed as:

(4) $$\begin{array}{c} J_{cw}\left(m\right)=Ae^{2\pi \left\{\left[\frac{B}{T_{LF}}{\left(mT_{s}-\left\lfloor \frac{mT_{s}-t_{0}}{T_{LF}}\right\rfloor +t_{0}\right)}^{2}\right]+fmT_{s}\right\}+\varphi } \end{array}$$

where *A* is the interfering signal amplitude; *B* and $T_{LF}$ represent the bandwidth and frequency modulation period of the chirp signal, respectively; $t_{0}$ is the time offset; and φ represents the phase.

## 3. The Proposed Method

**Figure 1:**
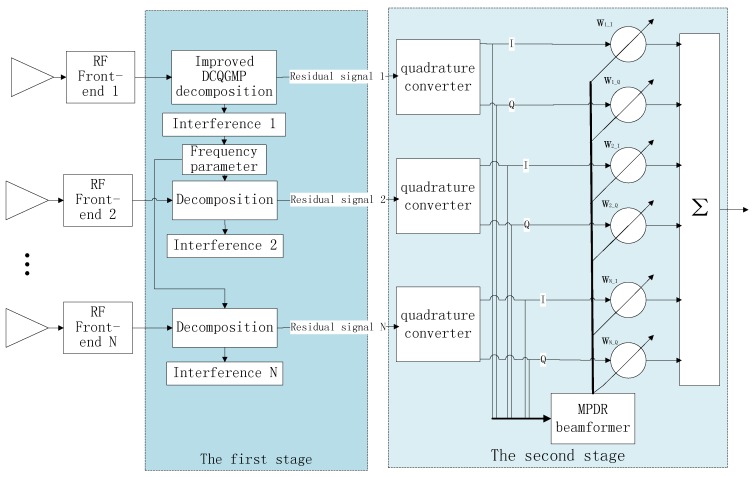
Block diagram of the proposed method.

In the coexistence of multiple types of interfering signals, there may be overlaps among different interfering signals in the time, frequency and spatial domains. How to deal with these interferences according to their different characteristics in different dimensions is an enormous challenge for existing methods. Focusing on this issue, a cascaded multi-type interferences mitigation method combining improved DCQGMP-based sparse decomposition and the MPDR beamformer is introduced, whose block diagram is shown in Figure 1. The signal model in Section 2 is a complex expression; however, there are only real signals in physical circuits. The signals in the RF front-end are real, and the in-phase (I) and quadrature (Q) signals obtained by using a quadrature converter usually are treated as the real part and imaginary part of the complex expression for array processing, respectively. Although it is feasible to suppress the CWI before or after the quadrature converter since the envelopes of CWI signals are both continuous in the two parts, the calculation of detecting and canceling CWI before the quadrature converter is half of doing that after the quadrature converter. Accordingly, the CWI signals’ detection and suppression are applied before the orthogonal transformation. In the first stage as shown in Figure 1, the received signals are sparsely decomposed to excise interfering signals, which can be sparse represented in the over-complete dictionary. We re-design the over-complete dictionary according to the characteristics of CWI signals to make the sparse decomposition suitable for multi-type interferences mitigation, and the necessary conditions for CWI signal detecting are analyzed, especially when the power of the CWI signals is lower than that of the wideband Gaussian interferences. In order to reduce the computation amount of the traditional MP algorithm, the DCQGA is used for MP optimization. Additionally, an improved DCQGA with the characteristic of high search density, adaptive step-size updating is suggested to improve the convergence rate and computational precision. Then, on the basis of these, a multi-channel signal interference suppression method based on the improved DCQGMP and a ratio terminate principle are proposed. In the second stage, the spatial DoF of the antenna array is employed to nullify the residuary interferences (such as Gaussian noise interferences) by utilizing the MPDR beamformer.

### 3.1. Multi-Channel Signal Interference Suppression Method Based on Improved DCQGAMP

#### 3.1.1. Matching Pursuit Decomposition

The MP algorithm, dealing with the signal sparse decomposition effectively, is an iterative “greedy” algorithm [21]. Compared with other sparse decomposition algorithms, it has flexibility and adaptability to signals. The key idea of this algorithm is that at each iteration, the best atom is selected and regarded as one of the components of the sparse representation.

Assume that $f_{x}$ is the signal to be decomposed, and define $D={\left(g_{r_{i}}\right)}_{r_{i}\in \Gamma }$ as the over-complete dictionary of functions, where $∥g_{r_{i}}∥=1$ is an atom and Γ is the parameter set of atoms. The inner product of $g_{r}$ and $f_{x}$ is defined as the similarity measure function *Z*, i.e., $Z\left(g_{r},f_{x}\right)=\langle g_{r},f_{x}\rangle$. After *l* iterations, we will get a codebook $B=\left\{b_{l}|b_{l}=\left(z_{l},g_{r_{l}}\right),z_{l}=Z\left(f_{x_{l}},g_{r_{l}}\right),l=1,2,\ldots ,L\right\}$. Additionally, the basic implementation steps of MP are as follows:

1. Initialization: l=0; $f_{x_{l}}=f_{x}$; B=⌀.
2. Compute the inner products $Z\left(g_{r},f_{x_{l}}\right)$ for all atoms; find the maximum amongst all inner products: $r_{n}=argmax_{g_{r}\in D}\left(Z\left(g_{r},f_{x_{l}}\right)\right)$; and add $b_{n}=\left(z_{l},g_{r_{l}}\right)$ to codebook *B*.
3. Compute the residual signal: $f_{x_{l+1}}=f_{x_{l}}-Z\left(g_{r_{l}},f_{x_{l}}\right)g_{r_{l}}$; if precision is reached, then stop; otherwise l=l+1, and iterate to Step 2.

If the termination condition is reached after the *L* steps’ decomposition, the signal can be expressed as:

(5) $$\begin{array}{c} f_{x}=\sum_{l=0}^{L-1}z_{l}g_{r_{l}}+f_{x_{L}} \end{array}$$

where $f_{x_{L}}$ is the residual signal.

#### 3.1.2. Analysis of Interference Detection Performance

In order to facilitate the analysis, only one single-tone or linear chirp interfering signal is considered in the following analysis, and assume that there are K2(K2>0) wideband Gaussian noise interfering signals. The GNSS signals are able to be ignored due to their relatively weak energy. Then, without considering the array steering vector, for *M* snapshots, at the *n*-th antenna, the signal before the quadrature frequency conversion can be written as:

(6) $$\begin{array}{c} \underset{M\times 1}{x_{r_{n}}}=\underset{M\times 1}{J_{cw\_r}}+\underset{M\times 1}{ℵ} \end{array}$$

in which:

(7) $$\begin{array}{c} \begin{array}{c} J_{cw\_r}\left(m\right)=\sqrt{p}\cos\left(fre\left(mT_{s}\right)+\varphi \right)\;\\ =\sqrt{p}\cos\left(2\pi \left\{\left[\frac{B}{T_{LF}}{\left(mT_{s}-\left\lfloor \frac{mT_{s}-t_{0}}{T_{LF}}\right\rfloor +t_{0}\right)}^{2}\right]+fmT_{s}\right\}+\varphi \right) \end{array} \end{array}$$

where *p* is the energy of the CWI signal and fre(•) represents the frequency parameter.

(8) $$\begin{array}{c} ℵ\left(m\right)=\sum_{i=0}^{K2}G_{i}\left(mT\right)+Re\left(\eta \left(mT\right)\right) \end{array}$$

where $G_{i}\left(•\right)$ is the *i*-th Gaussian interfering signal with zero mean and $\sigma_{i}$ variance and $Re\left(•\right)$ represents the real part of the complex expression. Then, **ℵ** can be regarded as a Gaussian noise with zero mean and σ variance.

(9) $$\begin{array}{c} \sigma =\sum_{i=0}^{K2}\sigma_{i}+\sigma_{\eta } \end{array}$$

Suppressing interferences using MP-based sparse decomposition can be considered as a process by which the received signal is adaptively decomposed in an over-complete dictionary of functions, providing a sparse linear expansion of CWI signals and the residual signal in which there is no CWI. The detectability of the interference signal affects the performance of the method, which is mainly determined by the over-complete dictionary, the interfering signal energy and the number of snapshots.

The over-complete dictionary should be constructed according to the broadband of desired signals and the prior knowledge about the types of interfering signals that can be obtained by experience or other investigative means. The real representation of CWI signals can be expressed by a cosine model with bandwidth, modulation period, initial frequency, fixed frequency and phase parameters. Hence, the over-complete dictionary should contain the corresponding parameter space. Most simply, select a series of linear frequency-modulated signals as atoms in the over-complete dictionary, which could be expressed as:

(10) $$\begin{array}{c} \begin{array}{c} g_{r_{i}}\left(m\right)=c_{r_{i}}\cos\left(fre_{r_{i}}\left(mT_{r_{i}}\right)+\varphi_{r_{i}}\right)\;\\ =c_{r_{i}}\cos\left(2\pi \left[\frac{B}{T_{r_{i}}}{\left(mT_{s}-\left\lfloor \frac{mT_{s}+t_{r_{i}}}{T_{r_{i}}}\right\rfloor T_{r_{i}}+t_{r_{i}}\right)}^{2}+f_{r_{i}}mT_{s}\right]+\varphi_{r_{i}}\right) \end{array} \end{array}$$

where $c_{r_{i}}$ is the normalized coefficient; $fre_{r_{i}}\in \left[f_{0}-\frac{B_{s}}{2},f_{0}+\frac{B_{s}}{2}\right]$, in which $B_{s}$ and $f_{0}$ represent the bandwidth and the center frequency of the atoms, respectively; $T_{r_{i}}$, $t_{r_{i}}$ and $f_{r_{i}}$ represent the frequency modulation period, the time offset and the initial frequency of the atom, respectively. The values of $k_{r}$, $t_{r_{i}}$, $f_{r_{i}}$ and $\varphi_{r_{i}}$ form the parameter set Γ, and they are selected at regular intervals according to their respective reasonable ranges and the number of atoms. The greater the number of atoms is, the higher the decomposition accuracy is.

The best atom is selected by looking for the maximum measure function *Z*:

(11) $$\begin{array}{c} Z\left(x_{r_{n}},g_{r}\right)=\langle x_{r_{n}},g_{r}\rangle =\langle J_{cw},g_{r}\rangle +\langle ℵ,g_{r}\rangle ={ℜ}_{J}+{ℜ}_{n} \end{array}$$

Assume that the best matching atom, $c_{r}cos\left(2\pi fre\left(mT_{s}\right)mT_{s}+\varphi \right)$, is in the over-complete dictionary and has been known. Then:

(12) $$\begin{array}{c} {ℜ}_{J}\left(\Delta fre,\Delta \varphi \right)=\sum_{m=0}^{M-1}\sqrt{p}\cos\left(fre\left(mT_{s}\right)+\varphi \right)c_{r}\cos\left(\left[fre\left(mT_{s}\right)+\Delta fre\left(mT_{s}\right)\right]+\left(\varphi +\Delta \varphi \right)\right)\;\\ =\frac{1}{2}c_{r}\sqrt{p}\sum_{m=0}^{M-1}\cos\left(\Delta fre\left(mT_{s}\right)+\Delta \varphi \right)\;\\ +\frac{1}{2}c_{r}\sqrt{p}\sum_{m=0}^{M-1}\cos\left(\left[2fre\left(mT_{s}\right)+\Delta fre\left(mT_{s}\right)\right]+\left(2\varphi +\Delta \varphi \right)\right) \end{array}$$

where Δfre and Δφ represent the frequency spacing and the phase spacing, respectively. Additionally, since *M* is large enough, we can suppose that the normalized coefficients, $c_{r}$, are equivalent to each other in the following analysis. In general digital signal processing, $T_{s}$ satisfies the Nyquist sampling theorem or the bandpass sampling theorem, and *M* is large enough, so the latter is much less than the former in the polynomial, then:

(13) $${ℜ}_{J}\left(\Delta fre,\Delta \varphi \right)\approx \frac{1}{2}c_{r}\sqrt{p}\sum_{m=0}^{M-1}\cos\left(\Delta fre\left(mT_{s}\right)+\Delta \varphi \right)$$

Additionally, the maximum value of ${ℜ}_{J}$ is the inner product of the signal to be decomposed, and the best matching atom:

(14) $${ℜ}_{J}^{max}\approx \frac{M}{2}c_{r}\sqrt{p}$$

In addition,

(15) $${ℜ}_{n}\left(\Delta fre,\Delta \varphi \right)=\sum_{m=0}^{M-1}c_{r}\cos\left(\Delta fre\left(mT_{s}\right)+\Delta \varphi \right)ℵ\left(mT\right)$$

Since **ℵ** is the Gaussian noise with zero mean and σ variance, then ${ℜ}_{n}$ is the Gaussian noise with zero mean and $Mc_{r}^{2}\sigma$ variance. Additionally, about 99.997 percent of the values drawn from a normal distribution are within four standard deviations $c_{r}\sqrt{M\sigma }$ away from the mean. Therefore, the necessary condition for detecting CWI signals is that the number of samples *M* should satisfy:

(16) $${ℜ}_{J}^{max}>4c_{r}\sqrt{M\sigma }$$

then, that is:

(17) $$M>64\frac{\sigma }{p}$$

#### 3.1.3. The Mutual Influence of Multiple Signals

In the previous section, we have analyzed the interference detection performance of the MP-based sparse decomposition when there is one interfering signal. However, there may be multiple signals to be dealt with. Then, taking the scenario that there are two interfering signals as an example, we analyze the effects on the method due to the presence of multiple signals. The two interfering signals can be written as:

(18) $$\begin{array}{c} \begin{array}{c} J_{cw\_r1}\left(m\right)=\sqrt{p_{1}}\cos\left(fre_{1}\left(mT_{s}\right)+\varphi_{1}\right) \end{array} \end{array}$$

(19) $$\begin{array}{c} \begin{array}{c} J_{cw\_r2}\left(m\right)=\sqrt{p_{2}}\cos\left(fre_{2}\left(mT_{s}\right)+\varphi_{2}\right) \end{array} \end{array}$$

where $p_{1}$ and $p_{2}$ are the energy of Interfering Signals 1 and 2, respectively; $fre_{1}\left(•\right)$ and $fre_{2}\left(•\right)$ represent the frequency parameter of the two interfering signals, respectively.

1. When $fre_{1}=fre_{2}$, the two interfering signals can be treated as one interference, and the reason is:
  (20) $$\begin{array}{c} \begin{array}{c} J_{cw\_r1}\left(m\right)+J_{cw\_r2}\left(m\right)=\sqrt{p_{1}}\cos\left(fre_{1}\left(mT_{s}\right)+\varphi_{1}\right)+\sqrt{p_{2}}\cos\left(fre_{2}\left(mT_{s}\right)+\varphi_{2}\right)\;\\ =\sqrt{p_{1}+p_{1}-2\sqrt{p_{1}p_{1}}\cos\left(\varphi_{1}+\varphi_{2}\right)}\cos\left(fre_{1}\left(mT_{s}\right)+\varphi_{t}\right) \end{array} \end{array}$$
  where $\varphi_{t}=arctg\left(\frac{\sqrt{p_{1}}\cos\left(\varphi_{1}\right)+\sqrt{p_{2}}\cos\left(\varphi_{2}\right)}{\sqrt{p_{1}}\sin\left(\varphi_{1}\right)+\sqrt{p_{2}}\sin\left(\varphi_{2}\right)}\right)$; and $\mathrm{arctg}\left(•\right)$ is the arc tangent function.
2. When $fre_{1}\neq fre_{2}$, the best atom corresponding to Interfering Signal 1 should be $g_{r_{1}}\left(m\right)=c_{r_{1}}\cos\left(fre_{1}\left(mT_{s}\right)+\varphi_{1}\right)$, then the influence of Interfering Signal 2 on Interfering Signal 1 can be expressed as:
  (21) $$\begin{array}{c} \begin{array}{c} \kappa =\langle g_{r_{1}},J_{cw\_r2}\rangle =\sum_{m=0}^{M-1}c_{r_{1}}\sqrt{p_{2}}\cos\left(fre_{1}\left(mT_{s}\right)+\varphi_{1}\right)\cos\left(fre_{2}\left(mT_{s}\right)+\varphi_{2}\right)\\ =\frac{1}{2}c_{r_{1}}\sqrt{p_{2}}\sum_{m=0}^{M-1}\cos\left(fre_{1}\left(mT_{s}\right)+fre_{2}\left(mT_{s}\right)+\varphi_{1}+\varphi_{2}\right)\\ +\frac{1}{2}c_{r_{1}}\sqrt{p_{2}}\sum_{m=0}^{M-1}\cos\left(fre_{1}\left(mT_{s}\right)-fre_{2}\left(mT_{s}\right)+\varphi_{1}-\varphi_{2}\right)\\ =\frac{1}{2}c_{r_{1}}\sqrt{p_{2}}\kappa_{1}+\frac{1}{2}c_{r_{1}}\sqrt{p_{2}}\kappa_{2} \end{array} \end{array}$$
  According to the types of the interfering signals, $fre_{1}\left(mT_{s}\right)+fre_{2}\left(mT_{s}\right)$ and $fre_{1}\left(mT_{s}\right)-fre_{2}\left(mT_{s}\right)$ can be treated as a quadratic function or a linear function of the sampling time.
  - When $fre_{1}\left(mT_{s}\right)+fre_{2}\left(mT_{s}\right)$ and $fre_{1}\left(mT_{s}\right)-fre_{2}\left(mT_{s}\right)$ are the quadratic function of the sampling time, let $fre_{1}\left(mT_{s}\right)+fre_{2}\left(mT_{s}\right)=\alpha_{1}m^{2}+\beta_{1}m$ and $fre_{1}\left(mT_{s}\right)-fre_{2}\left(mT_{s}\right)=\alpha_{2}m^{2}+\beta_{2}m$, where $\left|\alpha_{1}\right|>\left|\alpha_{2}\right|$. Then:
    (22) $$\begin{array}{c} \begin{array}{c} \kappa_{1}=\sum_{m=0}^{M-1}\cos\left(\alpha_{1}m^{2}+\beta_{1}m+\varphi_{1}+\varphi_{2}\right)=\sum_{m=0}^{M-1}\cos\left(\alpha_{1}{\left(m+\frac{\beta_{1}}{2\alpha_{1}}\right)}^{2}-\frac{\beta_{1}^{2}}{4\alpha_{1}}+\varphi_{1}+\varphi_{2}\right)\;\\ =\cos\left(\varphi_{1}+\varphi_{2}-\frac{\beta_{1}^{2}}{4\alpha_{1}}\right)\sum_{m=0}^{M-1}\cos\left(\alpha_{1}{\left(m+\frac{\beta_{1}}{2\alpha_{1}}\right)}^{2}\right)\;\\ -\sin\left(\varphi_{1}+\varphi_{2}-\frac{\beta_{1}^{2}}{4\alpha_{1}}\right)\sum_{m=0}^{M-1}\sin\left(\alpha_{1}{\left(m+\frac{\beta_{1}}{2\alpha_{1}}\right)}^{2}\right) \end{array} \end{array}$$
    Similarly,
    (23) $$\begin{array}{c} \begin{array}{c} \kappa_{2}=\cos\left(\varphi_{1}-\varphi_{2}-\frac{\beta_{2}^{2}}{4\alpha_{2}}\right)\sum_{m=0}^{M-1}\cos\left(\alpha_{1}{\left(m-\frac{\beta_{2}}{2\alpha_{2}}\right)}^{2}\right)\;\\ -\sin\left(\varphi_{1}-\varphi_{2}-\frac{\beta_{2}^{2}}{4\alpha_{2}}\right)\sum_{m=0}^{M-1}\sin\left(\alpha_{1}{\left(m-\frac{\beta_{2}}{2\alpha_{2}}\right)}^{2}\right) \end{array} \end{array}$$
  - When $fre_{1}\left(mT_{s}\right)+fre_{2}\left(mT_{s}\right)$ and $fre_{1}\left(mT_{s}\right)-fre_{2}\left(mT_{s}\right)$ are the linear function of the sampling time, let $fre_{1}\left(mT_{s}\right)+fre_{2}\left(mT_{s}\right)=\beta_{3}m$ and $fre_{1}\left(mT_{s}\right)-fre_{2}\left(mT_{s}\right)=\beta_{4}m$, where $\left|\beta_{3}\right|>\left|\beta_{4}\right|$. Then:
    (24) $$\begin{array}{c} \begin{array}{c} \kappa_{1}=\sum_{m=0}^{M-1}\cos\left(\beta_{3}m+\varphi_{1}+\varphi_{2}\right)\\ =\cos\left(\varphi_{1}+\varphi_{2}\right)\sum_{m=0}^{M-1}\cos\left(\beta_{3}m\right)-sin\left(\varphi_{1}+\varphi_{2}\right)\sum_{m=0}^{M-1}\sin\left(\beta_{3}m\right)\\ =\cos\left(\varphi_{1}+\varphi_{2}\right)\frac{\sin\left(\left(M+\frac{1}{2}\right)\beta_{3}\right)-\sin\left(\frac{\beta_{3}}{2}\right)}{2\sin\left(\frac{\beta_{3}}{2}\right)}-\sin\left(\varphi_{1}+\varphi_{2}\right)\frac{-\cos\left(\left(M+\frac{1}{2}\right)\beta_{3}\right)+\cos\left(\frac{\beta_{3}}{2}\right)}{2\sin\left(\frac{\beta_{3}}{2}\right)} \end{array} \end{array}$$
    Similarly,
    (25) $$\begin{array}{c} \begin{array}{c} \kappa_{2}=\cos\left(\varphi_{1}-\varphi_{2}\right)\frac{\sin\left(\left(M+\frac{1}{2}\right)\beta_{4}\right)-\sin\left(\frac{\beta_{4}}{2}\right)}{2\sin\left(\frac{\beta_{4}}{2}\right)}-\sin\left(\varphi_{1}-\varphi_{2}\right)\frac{-\cos\left(\left(M+\frac{1}{2}\right)\beta_{4}\right)+\cos\left(\frac{\beta_{4}}{2}\right)}{2\sin\left(\frac{\beta_{4}}{2}\right)} \end{array} \end{array}$$

**Figure 2:**
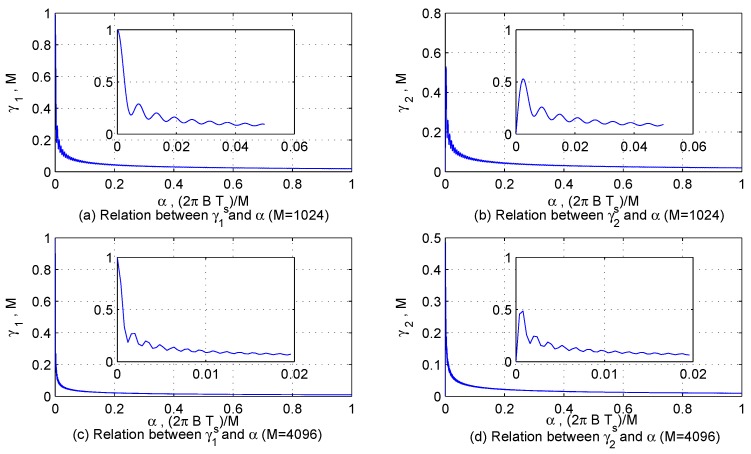
The features of $\gamma_{1}$ and $\gamma_{2}$.

**Figure 3:**
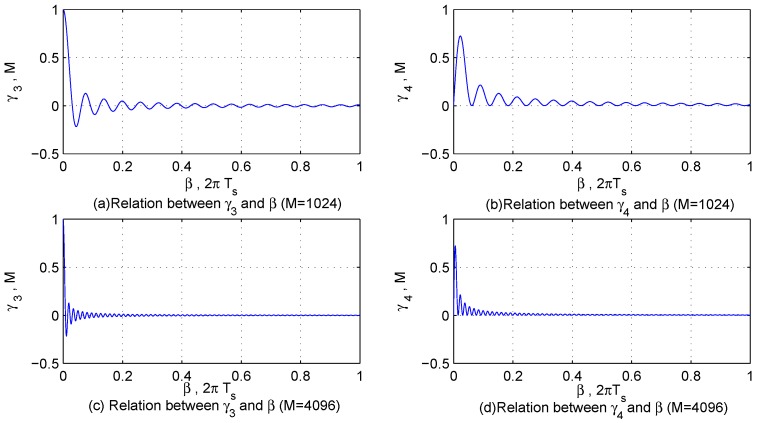
The features of $\gamma_{3}$ and $\gamma_{4}$.

In order to describe the characteristics of $\kappa_{1}$ and $\kappa_{1}$ more intuitively, let $\gamma_{1}=\sum_{m=0}^{M-1}\cos\left(\alpha {\left(m\right)}^{2}\right)$, $\gamma_{2}=\sum_{m=0}^{M-1}\sin\left(\alpha {\left(m\right)}^{2}\right)$, $\gamma_{3}=\frac{\sin\left(\left(M+\frac{1}{2}\right)\beta \right)-\sin\left(\frac{\beta }{2}\right)}{2\sin\left(\frac{\beta }{2}\right)}$ and $\gamma_{4}=\frac{-\cos\left(\left(M+\frac{1}{2}\right)\beta \right)+\cos\left(\frac{\beta }{2}\right)}{2\sin\left(\frac{\beta }{2}\right)}$, and their features are displayed in Figure 2 and Figure 3. According to the characteristics of interfering signals and the sampling theorem, $\left|\alpha_{1}\right|$ and $\left|\beta_{3}\right|$ are large enough to make $\kappa_{1}<<M$; however, $\left|\alpha_{1}\right|$, $\left|\beta_{2}\right|$ and $\left|\beta_{4}\right|$ may be so small that the value of $\kappa_{2}$ is similar to *M*.

In summary, the influence of Interfering Signal 2 on Interfering Signal 1 is mainly affected by the energy of Interfering Signal 2 and the difference of frequency parameters of the two signals. It shows a tendency to increase with the increase of the energy and the decrease of the difference of frequency parameters. From Equations (14), (23) and (25), it is found that we can reduce the influence of multiple interfering signals by increasing the number of sampling points *M*. Additionally, the conclusion can be extended to multiple interfering signals.

#### 3.1.4. The Improved MP Algorithm and Design Strategy of the Over-Complete Dictionary

In the MP-based decomposition, most of the calculation is spent on finding the best atom, and the number of atoms has a significant positive correlation with the number of parameters. Therefore, the less the parameters that should be obtained by the MP algorithm, the better. Fortunately, ${ℜ}_{J}$ is a periodic function over φ, and the period is 2π. In particular, ${ℜ}_{J}$ is a cosine function of φ, so φ can be obtained by the analytical method according to the periodicity and symmetry of ${ℜ}_{J}$ to reduce the the number of atoms. The detailed implementation way is as follows. Generate a group of atoms according to frequency parameters:

(26) $$\left\{\begin{array}{c} \begin{array}{c} g_{r_{i}\_1}=c_{r_{i}\_1}\cos\left(fre_{r_{i}}\left(mT_{s}\right)\right)\;\\ g_{r_{i}\_2}=c_{r_{i}\_2}\cos\left(fre_{r_{i}}\left(mT_{s}\right)+\frac{\pi }{2}\right)\;\\ g_{r_{i}\_3}=c_{r_{i}\_3}\cos\left(fre_{r_{i}}\left(mT_{s}\right)+\frac{\pi }{4}\right)\;\\ g_{r_{i}\_4}=c_{r_{i}\_4}\cos\left(fre_{r_{i}}\left(mT_{s}\right)+\frac{3\pi }{4}\right) \end{array} \end{array}\right.$$

Additionally, calculate the corresponding Δφ:

(27) $$\begin{array}{c} \Delta \varphi =\arctan\left\{\sqrt{2}\frac{\left|\langle x_{r_{n}},g_{r\_1}\rangle \right|+\left|\langle x_{r_{n}},g_{r\_2}\rangle \right|}{\left|\langle x_{r_{n}},g_{r\_3}\rangle \right|+\left|\langle x_{r_{n}},g_{r\_4}\rangle \right|}-1\right\} \end{array}$$

Then, let:

(28) $$\begin{array}{c} \varphi_{t}\in \left\{\Delta \varphi ,\;\Delta \varphi +\frac{\pi }{2},\;\Delta \varphi +\pi ,\;\Delta \varphi +\frac{3\pi }{2},\;\frac{\pi }{2}-\Delta \varphi ,\;\frac{3\pi }{2}-\Delta \varphi ,\;2\pi -\Delta \varphi \right\} \end{array}$$

Additionally, we can work out φ and the inner products corresponding to $fre_{r_{i}}$ as follows:

(29) $$\begin{array}{c} \varphi =\mathrm{argmax}\langle x_{r_{n}},c_{r_{i}}\cos\left(fre_{r_{i}}\left(mT_{s}\right)+\varphi_{t}\right)\rangle ,z_{r_{i}}=\langle x_{r_{n}},c_{r_{i}}\cos\left(fre_{r_{i}}\left(mT_{s}\right)+\varphi \right)\rangle \end{array}$$

#### 3.1.5. The Terminate Condition of MP

The conventional terminative condition of the MP algorithm is to judge whether the energy of the residual signal or the times of iterations meet the requirements. However, in the complicated electromagnetic environment, since the energy of the signals that can be sparse represented sometimes may be smaller than that of the other interferences, the energy of residual signal has little difference with the original signal energy; and because the number of signals that can have sparse representation is unknown, presetting the proper iterative number is also impossible. The existing terminative condition is not able to guarantee the effectiveness of the proposed method. Hence, a ratio terminate principle is introduced, which is to judge whether the ratio of the inner product of the best atom obtained and the signal to be decomposed in the *l* iteration to the variance of the residual signal meets the threshold. It can be expressed mathematically as:

(30) $$\begin{array}{c} \frac{\left|Z\left(g_{r_{l}},x_{r_{n_{l}}}\right)\right|}{\sqrt{M}c_{r_{l}}var\left(x_{r_{n_{l+1}}}\right)}=\rho \end{array}$$

where var(•) represents the variance function. Equations (16) and (17) show that when the signal to be decomposed contains the interference signal that can have sparse representation in the over-complete dictionary, inevitably there is a best atom making ρ>1.

#### 3.1.6. The Improved Double Chain Quantum Genetic Algorithm

Because the decomposition accuracy is related to the number of atoms, the size of the dictionary D is usually large. Additionally, in MP-based decomposition, most of the calculation is spent on finding the best atom. As a consequence, if we perform the MP algorithm in the usual way, the computation complexity will be very high. Therefore, we need to consider the fast algorithm for MP. One way to reduce the running time is to approach the optimization problem by using evolutionary computing. DCQGA is a probability optimization algorithm combining the quantum computation and genetic algorithm, with good global search capability and highly efficient parallel computing performance [25]. Another advantage of DCQGA is that its encoding space is continuous [26,27], which presents an effective approach to deal with continuous parameter spaces directly. Thus, the DCQGA can be used for the optimization problem in this paper, which will greatly reduce the amount of computation and improve the decomposition accuracy. Despite the effectiveness of DCQGA, it suffers from some disadvantages, such as: (1) the probability of searching for global optimum solutions is small due to the large coding space; (2) without considering the characteristics of the target function and the encoding mode, the conventional rotation angle computing function leads to low convergence speed and searching efficiency. To address that, a high density encoding mode and a cosine rotation angle computing function are proposed.

The conventional encoding method can be expressed as:

(31) $$\begin{array}{c} P_{i}=\left[\begin{array}{cccc} \cos\left(t_{i1}\right) & \cos\left(t_{i2}\right) & \ldots & \cos\left(t_{in}\right)\\ \sin\left(t_{i1}\right) & \sin\left(t_{i2}\right) & \ldots & \sin\left(t_{in}\right) \end{array}\right] \end{array}$$

where $t_{ij}=2\pi \times rd$, rd is a random number in [0,1]. i=1,2,…,n, j=1,2,…,m; *n* and *m* represent the colony size and the number of qubits, respectively.

It is noticed that the probability amplitude changes periodically, and the same solution appears two times in each gene chain. In other words, the traditional encoding method increases the number of global optimal solutions and sub-optimal solutions at the same time. Additionally, the increased number of sub-optimal solutions is much more than that of global optimal solutions. To solve this problem, compressing the traditional encoding space, let $t_{ij}\in \left[0,\frac{\pi }{2}\right]$, then the range of probability amplitude, $\alpha_{i}=\left|\cos\left(t_{i1}\right)\right|$ and $\beta_{i}=\left|\cos\left(t_{i1}\right)\right|$, is [0, 1]. In order to avoid that the probability amplitude value is not within [0, 1] when the phase exceeding the set range in the process of evolution, the encoding mode is improved as:

(32) $$\begin{array}{c} P_{i}=\left[\begin{array}{cccc} |\cos\left(t_{i1}\right)| & |\cos\left(t_{i2}\right)| & \ldots & |\cos\left(t_{in}\right)|\\ |\sin\left(t_{i1}\right)| & |\sin\left(t_{i2}\right)| & \ldots & |\sin\left(t_{in}\right)| \end{array}\right] \end{array}$$

Each of probability amplitudes corresponds to an optimization variable in solution space. If the *j*-th qubit on chromosome $p_{i}$ is ${\left[\alpha_{i}^{j},\beta_{i}^{j}\right]}^{T}$, the corresponding variables in solution space $\Omega =[a_{i},b_{i}]$ can be computed as follows:

(33) $$\begin{array}{c} X_{ic}^{j}=\alpha_{i}\left(b_{i}-a_{i}\right)+a_{i} \end{array}$$

(34) $$\begin{array}{c} X_{is}^{j}=\beta_{i}\left(b_{i}-a_{i}\right)+a_{i} \end{array}$$

Compared with the traditional encoding mode, although the reduction of the encoding space reduces the number of optimal solutions, it does not reduce the search probability of the optimal solution. On the contrary, the probability of finding the optimal solution is improved, and it is proven in the following analysis.

Let ϵ be an arbitrarily small positive constant. The solution space can be divided into $\frac{1}{2\epsilon }$ subintervals. When the solution falls in the subinterval that contains the global optimum solution, we can think that the global optimal solution with precision of ϵ is obtained. Without loss of generality, assume that there is one global optimum solution and $N_{g}$ chromosomes. According to the traditional encoding method, the probability of obtaining the global optimal solution can be expressed as:

(35) $$\begin{array}{c} P_{c}\left(i\right)=1-\frac{\left(1-N_{g}\epsilon \right)\left[1-\left(N_{g}+1\right)\epsilon \right]}{1-\epsilon } \end{array}$$

According to the proposed encoding method, the probability of obtaining the global optimal solution can be expressed as:

(36) $$\begin{array}{c} P_{p}\left(i\right)=N_{g}\epsilon \end{array}$$

Then, $P_{p}\left(i\right)>P_{c}\left(i\right)$. In other words, in the evolution of each generation, the probability of the improved DCQGA is higher than that of the traditional one to obtain the global optimal solution with precision of ϵ.

The conventional rotation angle computing function is written as:

(37) $$\begin{array}{c} \delta =e^{\nabla \hbar } \end{array}$$

where ∇ℏ is the changing trend of fitness function defined by Equations (12)–(14) in [23].

The exponential function can not fit the change trend of the cosine/sine encoding function well, so replace it with a cosine function; the adaptive cosine rotation angle computing function is written as:

(38) $$\begin{array}{c} \delta =\sin\left(\nabla \hbar *\frac{\pi }{2}\right) \end{array}$$

#### 3.1.7. The Multi-Channel Signal Interference Suppression Method Based on Improved DCQGMP

**Figure 4:**
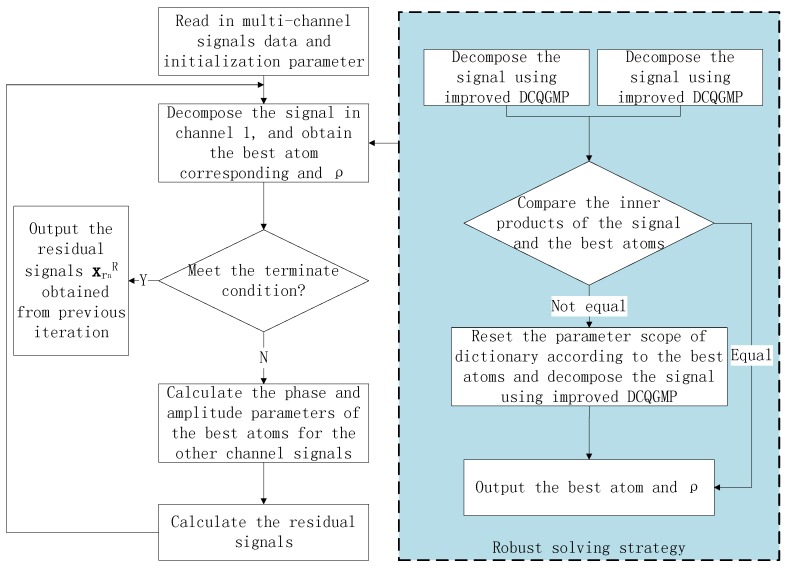
Frame of the multi-channel signals interference mitigation method based on improved DCQGMP.

Because the GNSS receiver used in the proposed method is equipped with an array antenna, there are multi-channel signals to be dealt with. If these signals are decomposed independently, the calculation is very great. Fortunately, the array signal processing theory demonstrates that there is a potential correlation among the multi-channel signals. In particular, they have the same frequency, similar amplitude and distinct phases. Therefore, we propose a multi-channel signal interference suppression method based on the improved DCQGMP. Its rough flow chart is shown in Figure 4. Since the phase parameter in each channel can be obtained by an analytical method, only one channel signal should be decomposed. Then, the phase parameters of signals in different channels are obtained according to the frequency parameters. Finally, the continuous wave interference signal is canceled.

As shown in the dashed box of Figure 2, in order to avoid that the improved DCQGMP algorithm converges to a local optimal solution, a robust solving strategy is used. Firstly, the signal is decomposed using two improved DCQGMP whose initial population and evolution are independent of each other. Additionally, the fitness function is:

(39) $$\begin{array}{c} z_{r_{i}}=\langle x_{r_{n}},c_{r_{i}}\cos\left(fre_{r_{i}}\left(mT_{s}\right)+\varphi \right)\rangle \end{array}$$

Secondly, compare their best fitness values. If they are equal, output the best atom and ρ; if not, select the atom with the larger fitness value as a suboptimal atom and come to the next step. Thirdly, reduce the search ranges of the parameters as $\frac{1}{100}$ of the original centered in the suboptimal atom, and decompose the signal again. Then, output the result.

### 3.2. MPDR Beamformer

In the second stage, the spatial filtering is applied, which can reject interferences and protect the GNSS signal by pointing the beam of receiver antenna array towards the GNSS satellite and away from interferers. In GNSS applications, the MPDR beamformer is one of the most powerful methods due to its effectiveness for interference suppression without considering the structure and direction of interfering signals. Additionally, the optimization problem for the space-only MPDR beamformer can be written as:

(40) $$\begin{array}{c} \min_{w}\;w^{H}\underset{N\times N}{R}w\;s.t.\;w^{H}a=1 \end{array}$$

where **w** represents the array weight vector; **a** is defined by Equation (2); R denotes the spatial covariance matrix of the residual signals obtained by Stage 1, which can be expressed by:

(41) $$\begin{array}{c} R=\frac{1}{M}X^{H}X \end{array}$$

where ‘*H*’ denotes conjugate transpose, and *M* is the size of snapshot data; $X=\left[{\left(x_{1}^{R}\right)}^{T}\;{\left(x_{2}^{R}\right)}^{T}\;\ldots \;{\left(x_{N}^{R}\right)}^{T}\right]$;

(42) $$\begin{array}{c} x_{n}^{R}=x_{r_{n}\_I}^{R}+x_{r_{n}\_Q}^{R} \end{array}$$

where $x_{r_{n}\_I}^{R}$ and $x_{r_{n}\_Q}^{R}$ are the in-phase (I) and quadrature (Q) signals obtained by using the quadrature converter. Then, the optimal weight vector is:

(43) $$\begin{array}{c} w_{\mathrm{opt}}=\frac{R^{-1}a}{a^{H}R^{-1}a} \end{array}$$

## 4. Simulation Results and Analysis

### 4.1. Performance of the Improved DCQGA

To show the performance of the improved DCQGA, an experiment of optimization of the function extreme is designed, and the improved DCQGA is compared with F-DCQGA [27] and conventional DCQGA [23]. Shaffer’s F6 which is a multi-modal function can be expressed as:

(44) $$f\left(x,y\right)=0.5-\frac{\sin^{2}\left(\sqrt{x^{2}+y^{2}}\right)-0.5}{{\left(1+0.001\left(x^{2}+y^{2}\right)\right)}^{2}}$$

There is only one global maximum point and infinitely many local maximum points in the range of both self-variables, which are both in $\left(-100,100\right)$. The global maximum point and global maximum are (0,0) and one, respectively. When the function value obtained by optimization algorithms is more than 0.9903, we consider that the global maximum is obtained.

**Figure 5:**
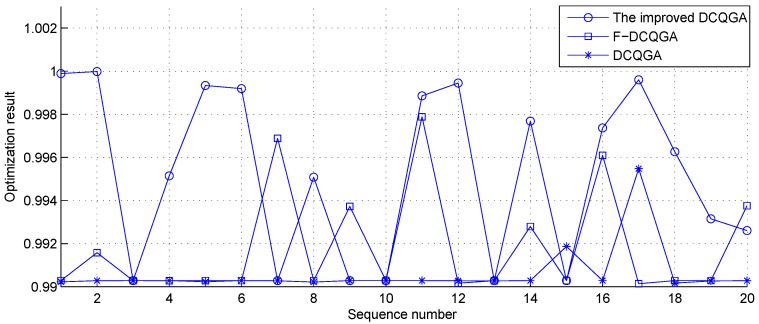
The comparison of the optimization results of Shaffer’s F6.

For the function mentioned, experiments are repeatedly executed 20 times, respectively, by the three optimization algorithms. The algorithm parameters are as follows: the population size is 50; the mutation probability is 0.1; the initial rotation angles are set to 0.005π, 0.01π and 0.05π (which are the best parameters obtained through simulations for each algorithm), respectively; the number of optimization steps is 100. The comparison of the optimization results is shown in Table 1 and Figure 3. From Table 1, we can find that although their running time is approximately equal, the improved DCQGA can be convergent with a higher probability and have a more accurate optimal solution than the other two methods. Combining Figure 5 and Table 1, it is obvious that the optimization result of the improved DCQGA is the best.

### 4.2. Performance of the Proposed Interference Mitigation Method

In order to assess the performance of the proposed method for interferences mitigation, three simulations have been conducted. In all simulations, a linear half-wavelength space antenna array with five elements is considered, and there is only one GNSS signal with a coarse acquisition (C/A) code rate of 1.023 MHz and multiple types of interferences. The first stage of the proposed method is operated on an intermediate frequency of 2.046 MHz, and the analog signal is sampled at 16.328 MHz. The parameters of improved DCQGMP and atoms are as follows: the population size is 1000; the mutation probability is 0.1; the initial rotation angles is set to 0.05π; the number of optimization steps is 40; $fre_{r_{i}}\in \left[1.046\;\mathrm{MHz},3.046\;\mathrm{MHz}\right]$, B=2MHz (or 0), $f_{r_{i}}\in \left[1.046\;\mathrm{MHz},3.046\;\mathrm{MHz}\right]$, $T_{r_{i}}\in \left[0.035\;\mathrm{ms},0.3\;\mathrm{ms}\right]$ and $t_{r_{i}}\in \left[0\;\mathrm{ms},T_{r_{i}}\right]$. Additionally, the termination threshold is set to 16. Then, the residual signals obtained by the multi-channel signals interference suppression method based on improved DCQGMP are down-converted to I and Q baseband signals using orthogonal frequency conversion technology. For both stages, the number of snapshots is cut to 24,492 points.

Since in MP-based sparse decomposition, most of the calculation is spent on computing the inner products of the signal to be decomposed and the atoms [21], we can adopt the number of inner products to approximately express the computational complexities of the conventional MP and the proposed DCQGMP. To ensure the performance of interferences suppression, the interval of $f_{r_{i}}$, $T_{r_{i}}$, $t_{r_{i}}$ and φ should not be more than 10 Hz, 10 ns, 10 ns and 0.01π, respectively. Additionally, the comparison of computational complexities is shown in Table 2. It illustrates that the computational complexity of the proposed DCQGMP is much less. In addition, since the DCQGMP can deal with continuous parameter spaces directly, we can achieve a higher decomposition accuracy.

The DOA of the GNSS signal with the signal to noise ratio (SNR) = −15 dB is $80^{\circ }$, and the Doppler frequency is 2 kHz. Additionally, the phase parameters of all interfering signals are randomly generated, and the other parameters are shown in Table 3.

#### 4.2.1. Influence of the DCQGMP-Based Interference Suppression on the GNSS Signal

**Figure 6:**
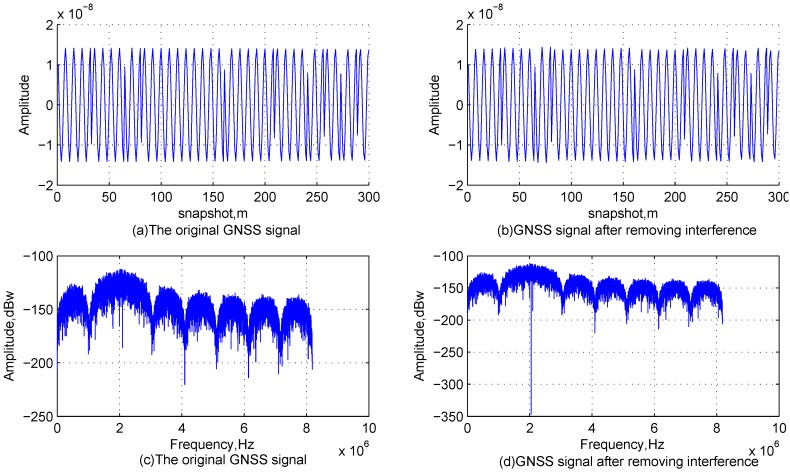
Characteristics of the GNSS signals for the first scenario.

**Figure 7:**
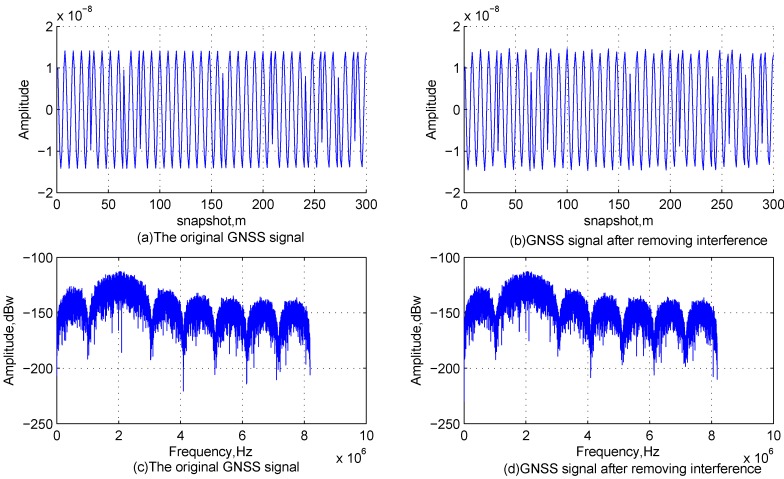
Characteristics of the GNSS signals for the second scenario.

To assess the influence of the DCQGMP-based interference suppression method on the GNSS signal, two experimental scenarios are considered. In the first scenario, only the GNSS signal and Interference 1 are present; Figure 6 shows the time-domain waveforms and frequency spectrum of the original GNSS signal and the GNSS signal after removing the interference. Comparing Figure 6c,d, it is obvious that only the energy at the interfering signal frequency is lost. From Figure 6a,b, we can hardly find the difference between the two signals, and the normalized mean square error (NMSE) of the GNSS signal is $2.208^{-10}$. In the second scenario, the GNSS signal is only contaminated with Interference 4. Additionally, Figure 7 shows that there is almost no difference between the original GNSS signal and the GNSS signal after removing the interference. Additionally, the NMSE of the GNSS signal after removing the interference is $4.162^{-10}$. Therefore, the effect due to the loss of the GNSS signal component in the dimension corresponding to interferences is very small.

#### 4.2.2. Performance of the Multi-Channel Signal Interference Suppression Method Based on Improved DCQGMP

In this simulation, to examine the interference suppression ability of the parallel multi-channel signal interference suppression method based on the improved DCQGMP introduced in Section 3.1.7, Interferences 1, 2, 4, 6 and 7 are considered. According to the analysis in Section 3.1, Interferences 1, 2 and 4 can be detected and canceled by the proposed method.

**Figure 8:**
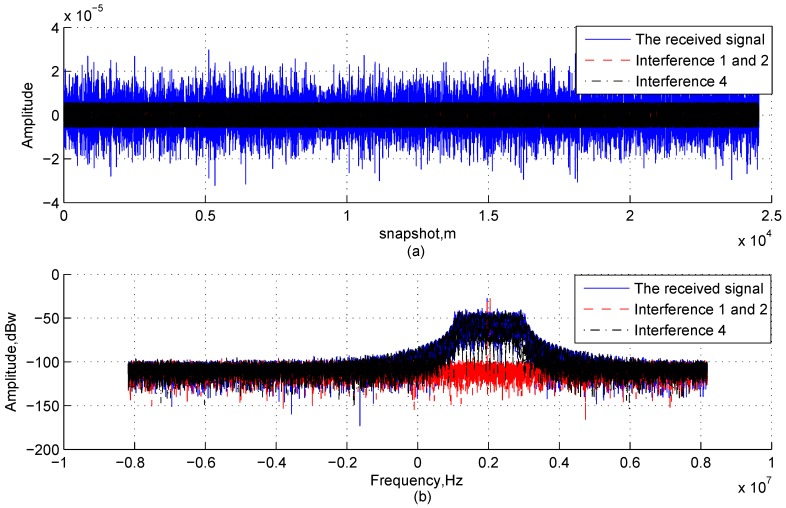
Characteristics of received signals in Channel 1.

**Figure 9:**
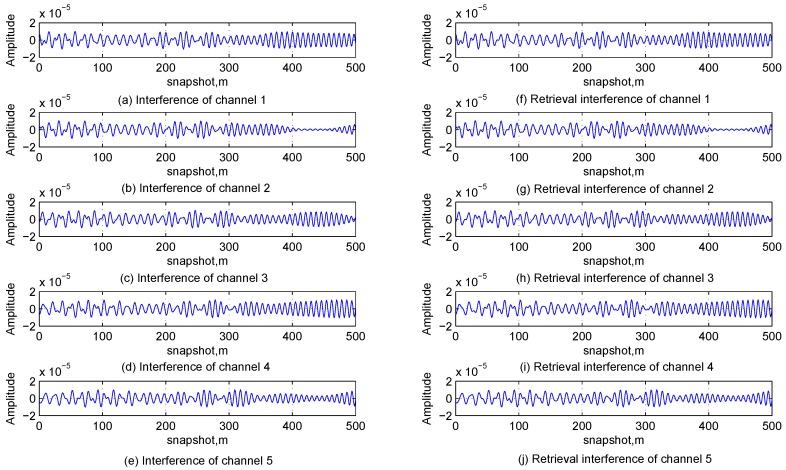
Time-domain waveform of original interferences and estimated interferences.

**Figure 10:**
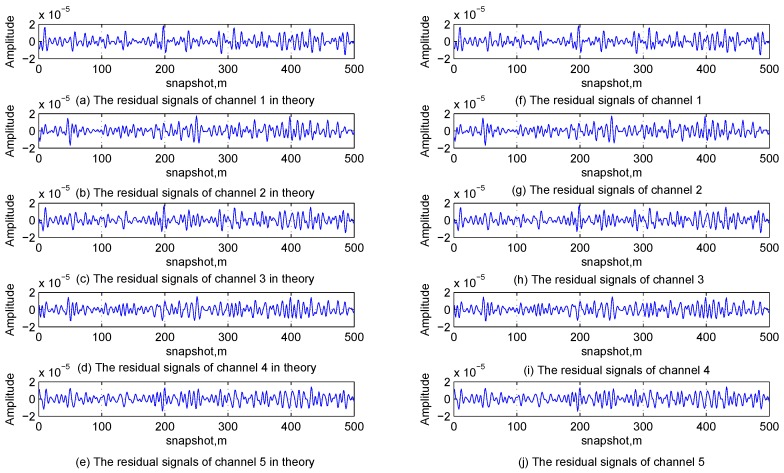
Time-domain waveform of residual signals in theory and estimated residual signals.

The time-domain waveforms and the frequency spectrum obtained from the FFT of the received signal in Channel 1 are shown in Figure 8a,b, respectively, and it is known from them that the multi-tone and linear chirp interfering signals are buried in Gaussian interferences and cannot be directly detected in both the time and frequency domains. Table 4 lists the relationship between the iteration number of the first stage and the terminate condition. It can be found that the values of ρ in the first three iterations are greater than the termination threshold, and it is consist with the analysis in Section 3.1.2, Section 3.1.3, Section 3.1.4 and Section 3.1.5 and the simulation conditions. Figure 9 shows the time-domain waveforms of original interfering signals and estimated ones; Figure 10 shows the time-domain waveforms of residual signals in theory and estimated residual signals by the proposed method. Comparing the waveforms of original signals and estimated ones, it can be found that the multi-channel signal interference suppression method based on improved DCQGMP can effectively detect and cancel the CWI from received signals. Additionally, the errors between these two set of signals can be measured with the NMSE, which are shown in Table 5. Accordingly, we can assume that there are no CWI signals in the residual signal.

#### 4.2.3. Performance of the Cascade Method for Multi-Type Interferences Mitigation

In this section, the performance of the proposed cascade method is compared with that of the well-known space-only MPDR (S-MPDR) beamformer, the space-time MPDR (ST-MPDR) [10] beamformer and the distortionless space-time adaptive (DST-MPDR) [16] processor. Additionally, four simulation scenarios are conducted. In Scenario 1, the number of interferences is less than that of antenna elements, and there is no interference with the same DOA as the GNSS signal. Therefore, Interferences 1, 4 and 6 are adopted. In Scenario 2, Interferences 3, 4 and 6 are used. In other words, there is one single-tone interfering signal with the same DOA as the GNSS signal. In Scenario 3, Interferences 2, 4, 6, 7, 8 and 9 are considered. This means that the number of wideband interferences is more than the spatial DoF of the antenna array. In Scenario 4, there is one linear chirp interfering signal with the same DOA as the GNSS signal and the number of wideband interferences is equal to that of antenna elements, and Interferences 2, 4, 5, 6, 7 and 8 are used. Additionally, the acquisition results obtained by coherent integration are employed to measure the performance of the methods for interference suppression. The integration time of the correlator is set to 1 ms.

**Figure 11:**
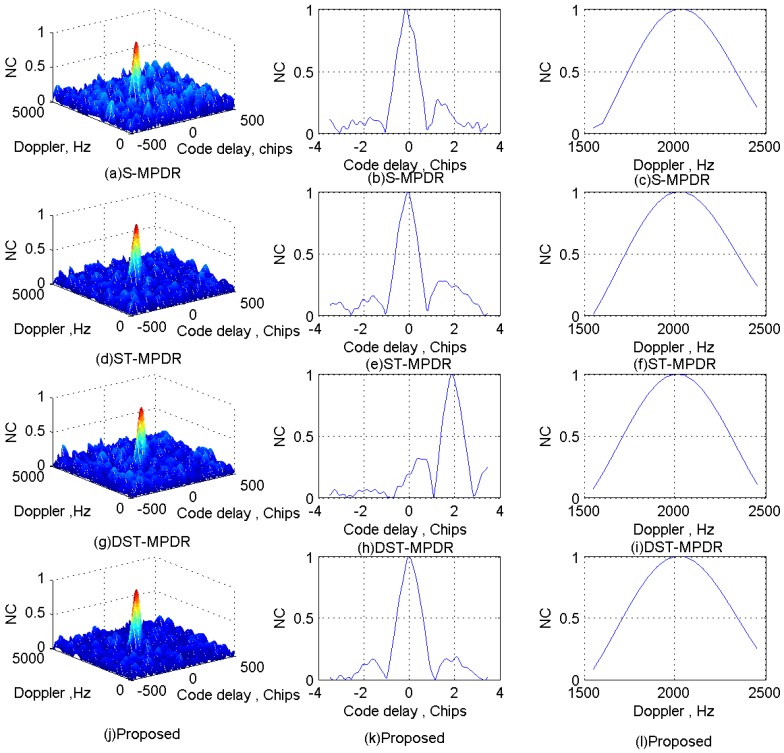
Correlation peaks after interference suppression for Scenario 1.

**Figure 12:**
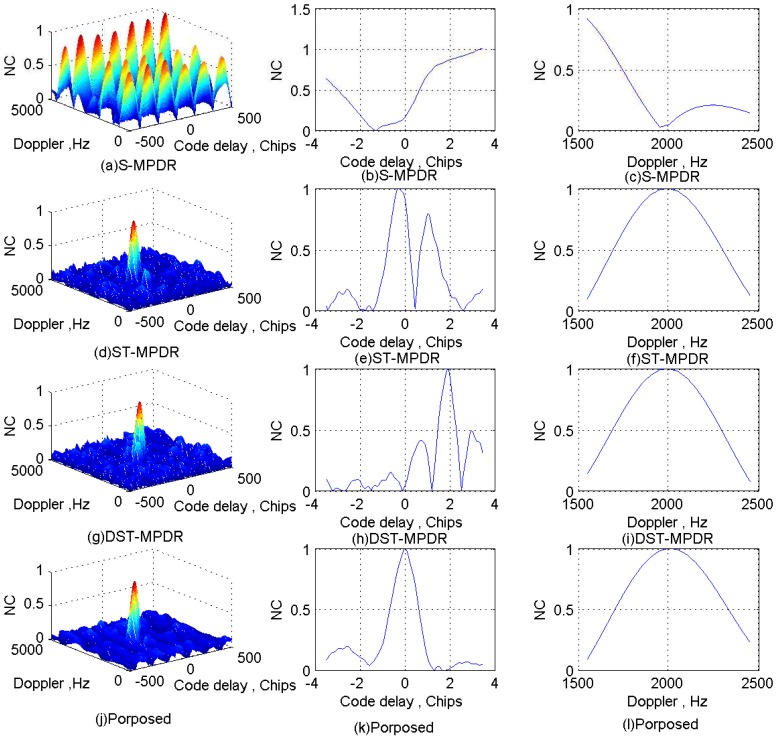
Correlation peaks after interference suppression for Scenario 2.

**Figure 13:**
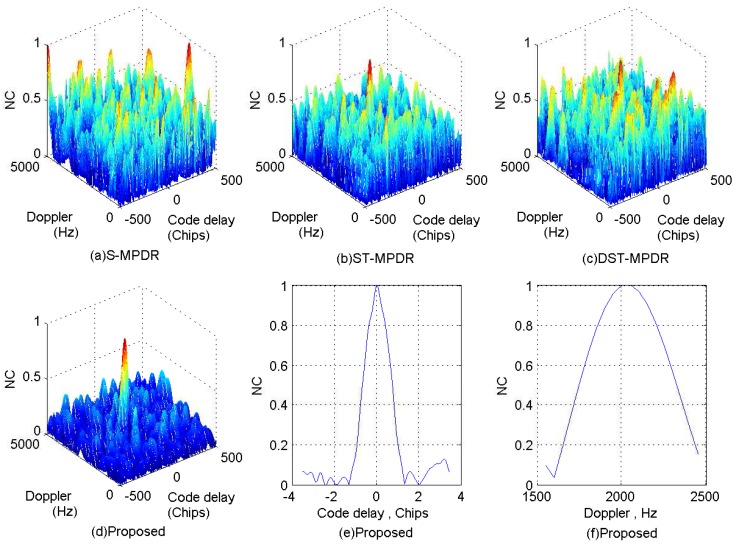
Correlation peaks after interference suppression for Scenario 3.

**Figure 14:**
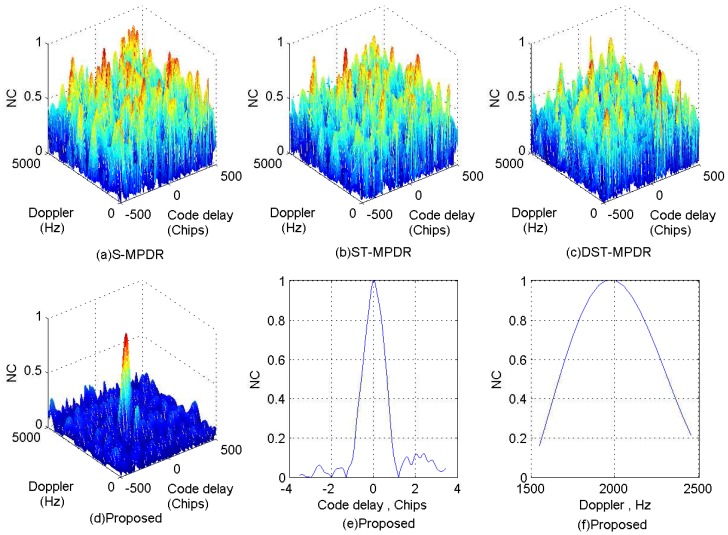
Correlation peaks after interference suppression for Scenario 4.

The normalized correlation (NC) peaks after interferences suppression using the four methods for each scenario are shown in Figure 11, Figure 12, Figure 13 and Figure 14. Additionally, Table 6, Table 7, Table 8 and Table 9 list the acquisition factor, which is the ratio of the maximum correlation value to the second maximum correlation value.

For Scenario 1, Figure 11 shows that all methods are effective to mitigate interferences and do not distort the NC peaks seriously. Additionally, from Table 6, we can find that by applying the proposed method, we can obtain the best acquisition factor.

For Scenario 2, Figure 12a–c shows that the S-MPDR beamformer failed to deal with the interferences due to the presence of the interfering signal arriving from the same direction as the GNSS signal; Figure 12d–f shows that although the ST-MPDR beamformer can suppress all interferences, it causes the distortion and shift of the correlation peak due to its nonlinear characteristics; Figure 12g–l shows that the DST-MPDR beamformer and the proposed method are still effective. Additionally, comparing Table 6 and Table 7, it is shown that the performance of the ST-MPDR beamformer and DST-MPDR beamformer degrades more seriously than that of the proposed method. In addition, from Figure 11h and Figure 12h, it is obvious that the DST-MPDR beamformer introduces code biases into the GNSS signal. However, the code phase bias introduced by DST-MPDR is pre-known and can be compensated [16].

For both Scenario 3 and Scenario 4, from Figure 13a–c and Figure 14a–c, it is obvious that the S-MPDR beamformer, ST-MPDR and DST-MPDR beamformer all failed to mitigate the interferences. This is because the number of wideband interferences is not less than the number of antenna elements or the DOA of some wideband interference is the same as the GNSS signal. Figure 13d–f and Figure 14d–f show that the shape and position of NC peaks are right. Additionally, Table 8 and Table 9 show that only by employing the proposed method, it is possible to acquire the GNSS signal. This is because that single-tone (multi-tone) and wideband linear chirp interfering signals can be canceled by sparse decomposition in the first stage.

## 5. Conclusions

Focusing on the complex electromagnetic environment in which multiple types of interfering signals coexist at the same time, a novel cascaded multi-type interferences mitigation method using sparse decomposition and array processing is introduced and examined in this paper. To solve the problem that there are overlaps among different interfering signals, the sparsity of the CWI signals and the advantage of the spatial processing are used. Firstly, the detectability of single-tone (multi-tone) and linear chirp interfering signals using the MP-based sparse decomposition is analyzed, and to reduce the running time of signal decomposition, an improved DCQGMP is introduced. Then, the multi-channel signals interference suppression method based on the improved DCQGMP is proposed, which can economize the spatial DoF of the antenna array by effectively detecting and canceling the single-tone (multi-tone) and linear chirp interfering signals even when they vanish into Gaussian interferences. Secondly, the MPDR beamformer is employed to mitigate the residuary interferences (such as Gaussian noise interferences) by utilizing the spatial DoF. Numerical simulations show that the proposed cascade method can not only suppress more interferences, but also does not significantly affect the position and shape of the correlation peaks of GNSS signals. Compared with the S-MPDR beamformer, ST-MPDR beamformer and DST-MPDR, the proposed method is able to deal with multi-type interferences more effectively and can suppress interferences with the same DOA as the GNSS signal, which can be sparse represented in the over-complete dictionary. Therefore, the proposed method can be implemented in the coexistence of multiple types of interfering signals to effectively improve the interference suppression performance of GNSS receivers while reducing the cost of space and hardware.

## Figures and Tables

**Table 1:** The comparison of their optimization results of Shaffer’s F6.

Algorithm	Best Result	Worst Result	Average Result	Convergence Times	Average Time (s)
Improved DCQGA	0.99998	0.99028	0.995266	14	0.07731
F-DCQGA	0.99788	0.99013	0.991806	7	0.07753
DCQGA	0.99547	0.99016	0.990603	2	0.07821

**Table 2:** Computational complexities of the proposed DCQGMP and the conventional MP.

Name	The Number of Inner Product for Single Channel Signal	Total Number of Inner Product
Conventional MP	$8.25\times 10^{10}$	$4.125\times 10^{11}$
Proposed DCQGMP	$2.64\times 10^{6}$	$2.64\times 10^{6}+44$

**Table 3:** The parameters of interfering signals.

Name	Type of Interference	Center Frequency (MHz)	Bandwidth (MHz)	DOA (°)	Interference to Noise Ratio (dB)	Others
1	Narrowband	2.046	0	60	25	/
2	Narrowband	1.962	0	130	25	/
3	Narrowband	2.08	0	80	25	/
4	Linear chirp	2.046	2	120	32	$T_{LF}=0.058$ ms $t_{0}=0.0012$ ms
5	Linear chirp	2.046	2	80	32	$T_{LF}=0.092$ ms $t_{0}=0.0095$ ms
6	Wideband Gaussian	2.046	2	20	32	/
7	Wideband Gaussian	2.046	2	35	32	/
8	Wideband Gaussian	2.046	2	165	32	/
9	Wideband Gaussian	2.046	2	135	32	/

**Table 4:** Relationship between the terminate condition and the iteration number.

Iteration Number	1	2	3	4
ρ	73	33.2	35.5	3.14
Whether to terminate	No	No	No	Yes

**Table 5:** Normalized mean square error (NMSE) of estimated signals.

Name	Channel 1	Channel 2	Channel 3	Channel 4	Channel 5
CW interference	0.0216	0.0190	0.0157	0.0235	0.0247
Residual signal	0.0182	0.0185	0.0132	0.0197	0.0207

**Table 6:** The acquisition factor for Scenario 1.

Name	S-MPDR	ST-MPDR	DST-MPDR	The Proposed
Acquisition factor	3.2	3.6	3.2	4.7
Peak’s position (code delay (chips), Doppler (Hz))	(0, 2000)	(0, 2000)	(1.875, 2000)	(0, 2000)

**Table 7:** The acquisition factor for Scenario 2.

Name	S-MPDR	ST-MPDR	DST-MPDR	The Proposed
Acquisition factor	1	1.26	2.0	4.0
Peak’s position (code delay (chips), Doppler (Hz))	/	(0, 2000)	(1.875, 2000)	(0, 2000)

**Table 8:** The acquisition factor for Scenario 3.

Name	S-MPDR	ST-MPDR	DST-MPDR	The Proposed
Acquisition factor	1	1	1	2.4
Peak’s position (code delay (chips), Doppler (Hz))	/	/	/	(0, 2000)

**Table 9:** The acquisition factor for Scenario 4.

Name	S-MPDR	ST-MPDR	DST-MPDR	The Proposed
Acquisition factor	1	1	1	2.5
Peak’s position (code delay (chips), Doppler (Hz))	/	/	/	(0, 2000)

## Abbreviations

The following abbreviations are used in this manuscript:

DCQGMP	Double chain quantum genetic matching pursuit
MPDR	Minimum power distortionless response
GNSS	Global navigation satellite system
MP	Matching pursuit
CWI	Continuous wave interference
DCQGA	Double chain quantum genetic algorithm
DoF	Degree of freedom
MVDR	Minimum variance distortionless response
STAP	Spatial-temporal adaptive processing
DOA	Direction of arrival
NMSE	Normalized mean square error
S-MPDR	Space-only minimum power distortionless response
ST-MPDR	Space-time minimum power distortionless response
DST-MPDR	Distortionless space-time minimum power distortionless response
NC	Normalized correlation
